# Tincture of Yellow Jessamine in the Treatment of Spasms, Fevers, Nervous Affections, Etc.

**Published:** 1901-07

**Authors:** N. F. Howard

**Affiliations:** Dahlonega, Ga.


### TINCTURE OF YELLOW JESSAMINE IN THE TREATMENT OF SPASMS, FEVERS, NERVOUS AFFECTIONS, ETC.

BY N. F. HOWARD, M.D.,

DAHLONEGA, GA.

I wish to say a few things in favor of tincture of yellow jessamine, as a medicinal agent, in the treatment of spasms, fits, nervous affections, fevers, etc. Many years ago I was called to see a little patient, the six or eight months' old infant of Mr. S., at the headwaters of the celebrated Yahoola river, ten miles north of Dahlonega.

I found the child suffering with spasms or fits peculiar to infants and children, and had been for some weeks. It had no intermission of the trouble, but would sit in its mother's lap and suffer with snapping of the eyes, drawing the face to one side, for hours. Then would come on a hard convulsion, which would last for several minutes, and then return to this partial remission as before. After this, in a few hours, another convulsion, and so on. I put the child under the treatment of tincture of gelsemium, eight or ten drops, to be given every three hours until the severity of these attacks should be broken up, the medicine to be discontinued when the severity of the fits was modified, but if they returned again in severity the medicine was to be given as before, but if the spells were light the medicine was to be reduced to five-drop doses. I also gave saccharate calomel, one-grain dose night and morning. In a very few days there was quite an improvement in the condition of the child, so that in the course of a month the child was entirely relieved of the spasms and remained free from these attacks, so far as I know.

I give another case. A few years ago a colored man, Fred Smith, brought his child, some eighteen months old, to my house one morning, who had been suffering all night and some days before with fits and spasms, without intermission. His eyes would snap and his face be drawn around to one side continually, and every few hours he would suffer with a fit or convulsion, and become rigid, remaining in this condition several minutes before relief would occur, and then he would suffer as before. I put him under the use of tincture of gelsemium, fifteen to twenty drops every three hours, the dose to be lessened if the severity of the attack was modified. I also gave him three grains of calomel at night and two grains of quinine twice a day. I am glad to say that in twenty-four hours his condition was very much improved, and in a week or two he was left free from the spasms. Yet when the medicine was left off he would have a light return of fits, which would subside as soon as the tincture was again given. Yet after a few weeks he seemed to have entirely recovered.

About eight years ago I treated a four-months-old infant, suffering with infantile spasms. I gave him tincture of gelsemium in six-drop doses, every three hours, half-grain doses of saccharated calomel night and morning, and one grain of phenacetin from time to time when the child had increased heat of the head. In a few days the little boy began to improve, and in two weeks seemed to be entirely relieved, and had not had a return of the trouble up to the present time.

If the editor will permit, I wish to relate some facts connected with this child's birth. In the midst of the labor the mother,

Mrs. H., was attacked with puerperal convulsions of the severest form. I sent for medical assistance. On consultation Dr. W. inserted under the skin in the arm five drops of Norwood's tincture of veratrum viride, of which she had already taken by the mouth the same amount. The severity of the convulsions was at once arrested. After this she suffered only with mild spasms, yet seemed to be, in a large sense, unconscious. One physician advised the removal of the infant by instruments. I found, on careful examination, that labor was progressing, and that effective pains were moving the infant on to a stage of certain delivery. We all rejoiced to see this effective. By waiting a time, therefore, we saved all risk from the use of instruments. The mother was restored at once to a safe condition, but the child seemed to be in a very hopeless condition, and none of us thought it could live, its head being in such a bruised state. But by proper care and attention it finally rallied and still lives.

At the close of the year 1865, I learned from Dr. M. H. Van Dyke that tincture of gelsemium was a very potent remedy in many forms of fever, as typhoid fever, scarlatina, measles, rose rash and most forms of nervous affections, especially for hysteria and nervous spasms, and nervous headache. The doctor had the utmost confidence in it, and gave it for nearly all forms of disease, and carried it with him wherever he went. I think it can be relied upon in spasms, convulsions and nervous affections. I have often given it in company with Dr. V. in typhoid fever, with good results. He would give it in doses of thirty drops every three hours to adults till the patient would see double, and then suspend it until this condition had passed off. We would find then the pulse changed to a soft, regular beat, decreased in number, the fever reduced from 103 to 100, the skin warm, the patient resting in a very pleasant state, and very often much improved.

We will remark here that the tincture to be effective must show a purple hue. If it does not show this color it is entirely unreliable, and will disappoint the physician every time.

I never saw any injurious effect from giving tincture of gelsemium, though I have seen many persons under its influence.

Dr. M. H. Van Dyke, a New Yorker by the way, but for a long time a citizen of Dahlonega, was with our army in '63 at Vicksburg, and at the battle of Baker's Creek, and fell into the lines of the enemy in our retreat.

Being in charge of the wounded he assisted in the amputation of the leg of Col. Skid Harris, of the 43d regiment, of which Dr. Van Dyke's son was a member. After this he was placed in charge of a hospital near, and treated the wounded. Near this place he met a German physician, from whom he obtained information that the tincture of yellow jessamine was a potent remedy in the treatment of fevers and other diseases, as stated in this paper.

Permit me to say I was much interested in hearing read in the April JOURNAL the prescriptions of different physicians for the treatment of colic in infants and children. Among all these prescriptions the one given by the editor in which the milk of assafetida was used is my favorite. A number of years ago I obtained information from my special friend and a successful physician in Gainesville, Ga., Dr. J. W. Bailey, that he depended for favorable results upon the same medicine, more than any other, for the treatment of colic in infants. Since then I have used the lac assafetida for colic in infants, generally with successful results.

